# Supplementary material for: The challenge of chromatin model comparison and validation: A project from the first international 4D Nucleome Hackathon
Source: PLoS Comput Biol. 2025 Aug 19;21(8):e1013358. doi: 10.1371/journal.pcbi.1013358 (PMC12380275; doi:10.1371/journal.pcbi.1013358)
Supplement: Attachment — Submitted filename: Response to Reviewers.docx [file pcbi.1013358.s004.docx]

*Dear Editor,*

*Thank you for the opportunity to submit a revised draft of our manuscript to PLoS Computational Biology. We appreciate the time and effort that the reviewers have dedicated to providing feedback on our manuscript. We have been able to incorporate changes to reflect the suggestions provided by the reviewers. We have highlighted the changes in the manuscript and outline these below.*

# Reviewer #1

Kubica et al. reported the findings of their analyses from the first 4D Nucleosome Hackathon, 2024. The authors designed workflows to benchmark five different computational methods for predicting 3D chromatin models from experimental Hi-C and ChIA-PET data sets. The authors compared the predictions at a specific 1Mb locus (chr1:178,421,513–179,491,193) in the GM12878 cell line. Since all the methods predicted 3D chromatin at different resolutions, the authors selected the predictions corresponding to 214 beads from the 1Mb window from all the methods. Here, each bead corresponds to approximately 5,000 base-pairs. The authors found the models trained using Hi-C, generated different predictions compared to the models trained using ChIA-PET. Furthermore, they compared the predictions across models trained using the same experimental data and stated that the predictions are variable across the five methods. Likewise, the authors compared the model predictions to experimental data from Hi-C, ChIA-PET, and SPRITE using Spearman correlation, and found that the models trained using a specific experimental data do not necessarily correlate with the given experimental data.

These analyses highlight the current limitations in existing methods for predicting 3D chromatin models. They also point out the need to understand the differences across the various experimental data sets as there is variability in the contact maps generated using different experimental protocols. Due to the lack of sufficient time for the hackathon, the benchmarking was done on a small set of methods at a single genomic locus. In future, it would be useful if such analyses were performed on a larger scale.

***Reviewer point #1:*** How were the five methods selected for benchmarking? The authors report these five methods as being user-friendly. Were these the only user-friendly methods or were there additional criteria for selecting these methods? What were the significant difficulties faced with each software in Table 1?

*Author response #1: We thank the reviewer for highlighting this important consideration. As suggested, we have included the criteria we tried to follow for selecting methods in the manuscript. We acknowledge in the revised manuscript that the methods ultimately selected for our project might not be the only ones satisfying our criteria. This introduces a level of arbitrariness, however, we hope that our results still contribute meaningfully to the discussion on the validation and use of 3D chromatin structure inference.*

*Difficulties we encountered during the hackathon included platform incompatibility, installation and dependency conflicts. This issue might be solved in the future by providing clearer documentation and/or tutorials, as well as containerization (e.g., Docker or Singularity).*

***Reviewer point #2:*** What were the default resolutions for each of the five methods before selecting 214 beads?

*Author response #2: We thank the reviewer for raising this point. We highlight this in the manuscript as follows:*

*“The default resolutions for MiChroM, MultiMM, LoopSage and DIMES (Hi-C only) was 1000 base pairs per bead, whereas for PHi-C2 and DIMES (ChIA-PET) it was 5000 base pairs per bead.”*

***Reviewer point #3:*** In Fig. 6b, are the models trained on ChIA-PET and not Hi-C?

*Author response #3: We thank the reviewer for highlighting this oversight in our language. We have revised the figure description as follows:*

*“Fig. 6. Comparisons of heatmaps generated from Hi-C models with a) the highest correlation (DIMES vs MultiMM), and b) the lowest correlation (MiChroM vs PHi-C2). All four heatmaps correspond to the interpolated models (number of beads = 214) that were generated for the same region of interest (chr1:178.421.513–179.491.193) based on Hi-C data.”*

***Reviewer point #4:*** Can the Spearman correlations be split by distance between the genome contacts? For example, all contacts within 250kb, 250-500kb, etc.

*Author response #4: We sincerely appreciate the reviewer's insightful comment. Indeed, comparing correlations and other metrics across genomic windows of varying distances provides a broader perspective on correlations at different scales. This valuable approach has been incorporated into our analysis following the review. Furthermore, the metric discussed in HiC-Rep offers distance-stratified measurements, which can be more informative than simple Pearson or Spearman correlations.*

***Reviewer point #5:*** The authors point out that the models trained using ChIA-PET have the highest correlation with Hi-C instead of ChIA-PET. Is it possible that these methods were originally designed for Hi-C, so the default parameters work best for predicting Hi-C contacts?

*Author response #5: We thank the reviewer for raising this point. Indeed, it may be of interest for the reader to understand why the models trained on ChIA-PET have the highest correlation with Hi-C. Inspired by this comment, we have significantly expanded the section preceding the Discussion, where we now provide a more in-depth analysis of the potential factors underlying the reviewer's observation.*

*We have emphasized two key aspects: (1) the possibility that many models were originally designed for Hi-C data, rather than ChIA-PET, which may influence their performance, and (2) the inherent differences in the structural properties of these data types. Specifically, we discuss how variations in signal distribution, "sharpness," and "smoothness" between Hi-C and ChIA-PET interaction maps could contribute to the observed higher correlations between ChIA-PET-trained models and Hi-C matrices.*

*We hope that these additions provide a more comprehensive explanation and clarify the factors contributing to this effect.*

# Reviewer #2

Kubica et al. presented a hackathon project aimed at comparing and validating 3D chromatin models. 3D model comparisons and validations are of interest to the field, though several major issues need to be addressed beyond the current analysis results.

***Reviewer point #1:*** A more clearly stated focus could improve the manuscript. Right now, the title and the first part look like a review or benchmark of different scales of interactions and various prediction tasks, but the results are mostly focused on inferring the 3D distance based on Hi-C or ChIA-PET.

*Author response #1: We thank the reviewer for making this suggestion. We have extended the Abstract, Introduction and Discussion sections to include a clarified focus consisting of:*

1. *Give an overview of what is the current state of the 3D chromatin field.*
2. *An effort to run different models.*
3. *An effort to validate these models.*

*“During the hackathon, we focused on running five distinct software packages and validating the models. Here we give an overview of current state of the 3D chromatin field and we discuss our efforts to run and validate the models.”*

*“The hackathon offered an opportunity to overview the current state of the 3D chromatin modelling field, as well as to define criteria for chromatin model comparison and validation. Although defining such criteria might seem conceptually straightforward (based on principles from polymer physics and statistical modeling), however, their implementation is highly complex and challenging due to the variability in experimental data and computational approaches. The objective of our hackathon project was to investigate different methodologies for chromatin modelling. This involved running five software packages, comparing their outputs to each other and experimental data. By testing different models, we aimed at identifying their strengths and limitations, as well as highlighting key challenges in model comparison and validation.”*

*“Here we provide an overview of what is the current state of the 3D chromatin field. We start with a literature review on available software and we show the variety of approaches for chromatin structure prediction, which leverage various experimental data types (e.g., , Hi-C, ChIA-PET, ChIP-seq, imaging data or a combination thereof) and assume different modelling principles. We list several software packages and classify them by the scale of modeling, starting from the smallest scale - loops and TADs, through chromosomes to the whole genome (Tables 1 and 2).”*

***Reviewer point #2:*** Only a general Spearman correlation is used to compare 3D distance results. As motivated in the first part of the manuscript, it would be interesting to assess 3D interactions in different scales.

*Author response #2: We appreciate the reviewer’s insightful comment. We acknowledge the limitations of using only Spearman correlation and have expanded our analysis to explore alternative validation metrics.*

*In our revised manuscript, we compare different metrics, including correlations at various resolutions and stratified distance metrics, to assess 3D interactions across scales. However, we emphasize that correlation-based metrics alone remain prone to biases, such as differences in smoothness and signal distribution. Importantly, comparisons with random models are essential—if a metric cannot distinguish between structured and random models, it is unreliable.*

*To address these issues, we tested distance correlations, KL divergence, and statistical tests, though most require significant preprocessing. One particularly promising metric is the Fréchet Inception Distance (FID), which consistently differentiates between biophysical and random models with minimal preprocessing, offering a more robust evaluation of model validity.*

***Reviewer point #3:*** Ensemble methods are supposed to mitigate the noise of the inference, whereas, in comparison, different methods have different choices of ensembling, which could drive the difference in comparisons. Thus, ensembling information need to be further investigated or controlled for in the comparison.

*Author response #3: We appreciate the reviewer’s insightful comment on ensembling methods, which are powerful for handling inference noise and interpreting dynamic chromatin patterns such as TAD borders and enrichment. In our work, we use LoopSage as a representative ensemble method, recognizing its value as a biophysical tool for understanding these patterns. However, validating such methods is complex due to their reliance on multiple biophysical hyperparameters, such as temperature in Monte Carlo simulations, sampling frequency, and factors governing loop extrusion dynamics. Different stochastic approaches, including Metropolis, Simulated Annealing variants, and Replica Exchange, require careful validation tailored to their specific mechanisms. In this revised version, we have expanded our discussion on these methods but retain the final structure of LoopSage as the most representative, ensuring a fair comparison with methods that produce a single structure.*

***Reviewer point #4:*** Another major issue is validation. Although the authors acknowledge it is hard to validate, it will be more informative if orthogonal information (e.g., image-based 3D distance measurements) can be used to compare with 3D models based on Hi-C and ChIA-PET. It is not clear to me how and why Hi-C-based distance is used to evaluate Hi-C-based 3D models’ distance.

*Author response #4: We sincerely appreciate the reviewer for raising this important point. We fully agree that orthogonal validation is crucial for ensuring the reliability of 3D models. However, we also note that some methods do incorporate Hi-C matrices as part of their validation strategies in the absence of available orthogonal image-based information (e.g. Carstens et al., 2016). In response to this, we have substantially expanded our discussion on validation approaches in the "Challenge of Model Validation" section. This revised section provides a more comprehensive overview of the validation methodologies commonly used in the field. We hope that these additions enhance the clarity and depth of our discussion, ensuring a more thorough treatment of 3D chromatin model validation.*

***Reviewer point #5:*** How does inference based on micro-C compare to inference based on Hi-C and ChIA-PET?

*Author response #5: Thank you for your insightful comment. While our study does not focus on micro-C, we recognize its relevance in the context of chromatin interaction inference. To address this, we have added a brief discussion in the introduction, highlighting some of the differences between inference based on micro-C, Hi-C, and ChIA-PET. We also mention a few studies in which micro-C data was used in the context of 3D chromatin structure inference. However, as our study primarily relies on Hi-C and ChIA-PET, we have maintained our focus on these techniques. We appreciate your suggestion and believe this addition strengthens the contextualization of our work.*

***Reviewer point #6:*** Currently, the comparison result is mostly limited to one cell line. Although the time is limited for a hackathon, it would be more conclusive if more cell lines were included.

*Author response #6: We appreciate the reviewer’s comment regarding this point. We agree that incorporating several cell lines would likely provide broader biological insights. Nevertheless, in this study, we primarily focus on comparing and validating chromatin models rather than exploring cell-type-specific chromatin structures. Moreover, given the time constraints of the hackathon, our aim was to establish a robust comparison framework rather than conduct an analysis using datasets with multiple cell lines. In the future, we might extend it to include several cell lines, but we believe that our findings are still impactful within the objective of this study.*

***Reviewer point #7:*** The color scheme is lacking, and the legends are the same for Figures 5 and 6.

*Author response #7: We thank the reviewer for the suggestion. In response, we have incorporated an appropriate color scheme and legends in Figures 4 and 5, ensuring that the modeled chromatin region is annotated and well-defined.*

# Reviewer #3

The authors report the challenge and results of one of the projects for 4D Nucleome Hackathon, aimed at benchmarking computational models of chromatin structure modeling. The authors highlight the complexity and hierarchical organization of chromatin, as well as the variety of modeling approaches and scales that contribute to the difficulty of model comparison and validation. They propose a workflow that uses Spearman correlations to quantify similarity between models and between models and experimental data. Their work underscores the importance of standardized guidelines and improved software practices to advance the field of 3D genome modeling. I have a few comments:

***Reviewer point #1:*** In Introduction, ChIA-PET and Hi-ChIP experiments produce contact matrices and prove efﬁcacious in TAD-scale modeling. Shouldn't ChIA-PET and Hi-ChIP are used mainly for detecting loops?

*Author response #1: We thank the reviewer for making this remark. We acknowledge that ChIA-PET and HiChIP data have primarily been utilized in the context of loop detection and modeling in previous research. However, we also highlight that several methods included in our study explicitly claim the ability to make use of either Hi-C, ChIA-PET, or HiChIP data for chromatin structure modeling—MultiMM being a notable example. In response to this feedback, we have revised the sentence with “TAD-scale modeling”, placing greater emphasis on the role of loop modeling when using ChIA-PET and HiChIP data. Additionally, we have expanded our discussion in the subsequent paragraphs to provide a clearer explanation of how different data types, including ChIA-PET, HiChIP, and Micro-C, contribute to 3D chromatin modeling. We hope that these modifications enhance the clarity and justification of our ChIA-PET data usage, making it more accessible and comprehensible to the reader.*

***Reviewer point #2:*** Related to point 1, ChIA-PET is used for identifying looping that involve specific proteins. The authors should specify which ChIA-PET data they are using (in this case I believe it's CTCF and SMC1A)

*Author response #2: We thank the reviewer for highlighting this oversight. We added the appropriate descriptions of the specific ChIA-PET datasets used in our study both in the Materials and Methods section as well as in the main text of the manuscript to specify which ChIA-PET we were using.*

***Reviewer point #3:*** In the introduction the author briefly touched upon single cell Hi-C, I think it's worth mentioning other single cell multi-omics with chromatin interactions included such as sn-m3c-seq, HiRES, GAGE-seq as they facilitate the training of predictive modeling at single cell resolutions. Related to this, SPRITE, ChIA-Drop and other potential multi-way interaction profiling method could also motivate new models being created.

*Author response #3: Thank you for an excellent suggestion. We agree with the comment that the other multi-omics as well as multi-way interaction profiling methods for capturing single-cell chromatin information are worth including in the introduction. Following the reviewer’s suggestion the “Methods for chromatin structure modeling” section of the introduction was greatly expanded. Besides the methods suggested by the reviewer, we have also added a short discussion of additional such methods and their potential use in future chromatin modeling. We hope that these revisions provide a more comprehensive overview of the field and enhance the clarity and breadth of our discussion.*

***Reviewer point #4:*** When discussing existing works, I think it's also worth to discuss existing evaluation metrics for these models as this paper is mainly about evaluation, discussing previous work on this part is crucial. For example, the Distance-Stratified Correlation Coefficients, softwares like GenomDISCO, HiCRep etc.

*Author response #4: We thank the reviewer for highlighting these highly relevant publications. We include the following text in the Discussion section to place our study in the context of this previous research:*

*“Currently, there is no robust statistical metric to evaluate the quality of the experimental data. For that reason, state-of-the-art frameworks such as HiCRep (Yang et al., 2017) or GenomeDISCO (Ursu et al., 2018) have been developing to assess the reproducibility of Hi-C that take into account the sensitivity to noise and the unique spatial features of the data, including domain structures and distance-dependence.”*

*We were excited to re-implement these metrics and try to adapt them in the problem of validations since both of them provide essential approaches for the heatmap comparison. GenomeDISCO provides an approach that takes advantage of random walk distances to resolve the problem with the heatmap smoothness, and HiC-Rep provides a stratified correlation metric that should in principle provide higher separation between different classes of data. Nevertheless, we acknowledge that these methods are made for comparisons between different experimental heatmaps, and not to compare simulated with experimental data, and thus it needs to be handled carefully. We provide a comparison of these metrics and other statistical tests. Finally, we would like to add another metric that was proven to be very helpful for the identification of simulated structures as more reliable than random structures, and this is the Réchet inception distance (FID) metric.*

***Reviewer point #5:*** I suggest for Table 1, split it into several tables based on if it's predictive modeling or polymer modeling etc. currently it's a long table and a mixture of different models.

*Author response #5: We sincerely appreciate the reviewer’s valuable suggestion. We agree that Table 1 was overly long and somewhat unclear, as it combined different classes of models. To improve clarity, we have split the original table into two separate tables. Table 1 now exclusively presents genomic data-driven models, including those designed for bulk Hi-C and single-cell modeling. Table 2 includes methods that did not fit neatly into Table 1, such as image-based approaches and models that generate 3D contact matrices rather than traditional 3D polymer reconstructions. We hope that these adjustments make the method overview clearer and more accessible to the reader.*
