# Supplementary material for: The challenge of chromatin model comparison and validation: A project from the first international 4D Nucleome Hackathon
Source: PLoS Comput Biol. 2025 Aug 19;21(8):e1013358. doi: 10.1371/journal.pcbi.1013358 (PMC12380275; doi:10.1371/journal.pcbi.1013358)
Supplement: Attachment — Submitted filename: Response_to_Reviewers_auresp_2.docx [file pcbi.1013358.s005.docx]

*Dear Editor,*

*Thank you for the opportunity to submit a revised draft of our manuscript to PLoS Computational Biology. We appreciate the reviewers’ time and thoughtful feedback. In response, we have made revisions to the manuscript in accordance with their suggestions. All changes have been highlighted in the manuscript and are outlined below.*

# Reviewer #1

Reviewer #1: The authors have addressed most of my comments. However, it is not clear whether they calculated Spearman correlations split by distance. In response to comments 1.4 and 2.2 they stated that they calculated different metrics based on distance stratifications but I was unable to find the related section and figures in the manuscript. Could the authors highlight the relevant sections? Additionally, calculating these metrics based on distance stratification is important because it would highlight which models are more accurate in predicting distal enhancers which are key elements of gene regulation.

*Author response #1: We sincerely thank the reviewer for this insightful comment. We apologize for the oversight — indeed, we had computed the stratified Spearman correlation metrics, but inadvertently omitted them from the manuscript. We have now included these results in the revised version, specifically in the section titled “Challenge of model validation”.*

*The updated analysis confirms that the genomic distance at which correlations are computed has a substantial impact: different modeling strategies show varying agreement depending on spatial scale. This highlights that models tend to perform best at the resolution they were designed for — for instance, a model optimized for TAD-scale structures may underperform at finer resolutions. This reinforces the idea that each model captures distinct aspects of chromatin architecture, reflecting the inherent variability in 3D genome reconstruction approaches.*

*We appreciate the reviewer’s feedback, which helped us improve the clarity and completeness of our analysis.*

# Reviewer #2

Reviewer #2: Thank you for your efforts in trying to address many of the points raised. I have several follow-up concerns on some of the points:

Regarding Reviewer 2, point 4, it is still concerning that the evaluation labels overlap with the input. This may weaken both the study design and the strength of the conclusions. The authors opted to retain the original validation approach, citing that “some methods do incorporate Hi-C matrices as part of their validation strategies in the absence of available orthogonal image-based information (e.g., Carstens et al., 2016).” However, this rationale seems problematic. In this setup, a model that simply reproduces the input Hi-C distances could achieve seemingly perfect performance, which would not be informative. Thus, orthogonal ways to evaluate seems key to draw informative conclusions.

Additionally, several critical analyses mentioned in the response letter do not appear to be included in the manuscript. Specifically, I was unable to locate the distance-based comparisons or the KL-divergence analyses brought up in Reviewer 1, point 4, and Reviewer 2, point 2.

*Author response #2: We thank the reviewer for the constructive comments. We agree that relying on Hi-C data for both input and evaluation has inherent limitations. While the Pearson correlation between input and reconstructed heatmaps is well below 1.0 — indicating that the models do not simply replicate the input — we acknowledge that this does not represent an orthogonal validation. However, it highlights how challenging it is even to recover the input data faithfully. We also note that comparing simulated and experimental heatmaps is not straightforward, as such comparisons often require transformations that can unintentionally distort the data and lead to unreliable conclusions. Some methods, such as LoopSage, address this by generating ensembles that better match contact maps; however, in our study, we used only one model per method to ensure a consistent and fair comparison, since most approaches did not provide ensembles.*

*We fully agree on the value of orthogonal validation. While image-based methods like FISH are often useful, we decided not to include them due to two main limitations: the lack of available data for our specific 1 Mb region, and the high variability and stochastic nature of such methods in this scale (~1 Mb). In our opinion, these make imaging more suitable for chromosome-wide assessments than for focused local modeling. Instead, we incorporated publicly available ChIA-PET data to validate loop structures from an independent source (Hi-C). We believe that the distinct principles and mechanisms employed by ChIA-PET and Hi-C methods provide a sufficient degree of orthogonality for a meaningful validation of our structures. Moreover, we used SPRITE data for validation as well, which is an additional orthogonal dataset. This allowed us to introduce a strong layer of validation within the practical constraints of the 5-day hackathon setting, where our goal was to explore and benchmark multiple modeling approaches quickly and within a narrow genomic window.*

*Finally, we wish to clarify that our intention was not to propose a definitive validation framework, but rather to reflect on the methodological and conceptual challenges of 3D chromatin modeling. Biophysical models are appropriate for testing mechanistic assumptions under specific conditions, rather than broad generalization, as is the case in machine learning. We also apologize for the omission of figures in the original submission—both the stratified distance analyses and KL-divergence metrics are now included in the manuscript. We are grateful for the reviewer’s careful reading and hope that our efforts to address these points clarify the intentions and limitations of our work.*

# Reviewer #3

Reviewer #3: The authors have addressed all my previous questions, I have no further comments
